# Distribution of Medical and Surgical History of the War of the Rebellion

**Published:** 1873-08

**Authors:** 


### Distribution of the Medical and Surgical History of the War of the Rebellion.

Complaint is very general as to the manner of the distribution of this truly valuable work. It appears that the public libraries, the Medical Journals and the Members of Congress received nearly the entire edition, and that it is impossible to obtain the work by direct purchase. The volumes given Congressmen were, of course, designed for judicious distribution in the several representative districts, Society Medical Libraries, Medical College Libraries, Hospital Libraries, and similar institutions having claims prior to private citizens, or the political supporters or personal friends of the representatives. It would be absurd to suppose that these volumes were given to Representatives for any other than judicious distribution; and still it appears that they are offered *for sale*, as we see by a New York paper, showing that "somebody" is offering to sell the volumes designed for appropriate gratuitous distribution. If the United States pay representatives in scientific and professional books and throw upon them the burden of sale, then there may be in this no just ground of complaint. It is to be greatly deprecated that so valuable a work cannot be obtained by purchase, if not otherwise, so that all physicians who desire can obtain it. The compilers of these volumes have accomplished a great work and the results of their labor should be placed in a position where the profession may profit by it.

